# China’s long march to malaria elimination: a case of adaptive management

**DOI:** 10.1186/s12936-021-04038-w

**Published:** 2022-02-08

**Authors:** Duo-quan Wang, Xiao-hui Liang, Shen-ning Lu, Wei Ding, Jing Huang, Xin Wen, Shan Lv, Ning Xiao, Lewis Husain, Xiao-Nong Zhou

**Affiliations:** 1 National Institute of Parasitic Diseases, Chinese Center for Disease Control and Prevention; Chinese Center for Tropical Diseases Research; WHO Collaborating Centre for Tropical Diseases; National Center for International Research on Tropical Diseases, Ministry of Science and Technology; Key Laboratory of Parasite and Vector Biology, Ministry of Health, Shanghai, China; 2 School of Global Health, Chinese Center for Tropical Diseases Research, Shanghai Jiao Tong University School of Medicine, Shanghai, China; 3 School of Public Health, Global Health Institute, Wuhan University, Wuhan, China; 4 Institute of Development Studies, Brighton, UK

**Keywords:** China, Malaria elimination, Adaptive, Global health

## Abstract

Since the 1950s, China has transitioned from a malaria pandemic country with tens of millions of annual cases, through phases of local control and elimination, to sustained national malaria elimination efforts. This marks the first time a country in the World Health Organization (WHO) Western Pacific region has been certified malaria-free in more than 3 decades. This article provides an innovative approach to understanding China’s malaria elimination journey. A number of articles and commentaries have analysed the effectiveness of specific technical approaches implemented in China. Our argument is that we need to look beyond these, and consider the ways in which policy development and implementation capacities have been fostered to support the dynamic change management. The article makes a number of arguments. First is the pragmatic adaptiveness of policies and strategies—and implementation capacities. Second, China has invested in building systems as well as capacities to support the elimination of parasitic diseases, including malaria. Third, the country has both benefited from, and contributed to, global health collaboration on malaria elimination. The ongoing work by the authors is identifying a number of key factors.

## Background

This is a landmark year for public health in China, as the country is declared free of malaria for the first time. As China joins the club of countries that have eliminated malaria, there is interest from the global health community—inside and outside China—in what has worked well in China and how China’s experience can help inform elimination efforts elsewhere. Malaria is a priority for the Chinese government in its global health cooperation programmes. This reflects the global need for such cooperation—there were over 140 million new infections and 0.4 million deaths in 2020, the majority in sub-Saharan Africa, and it is feared that COVID-19 will significantly set back malaria control and elimination efforts [1].

**Fig. 1:**
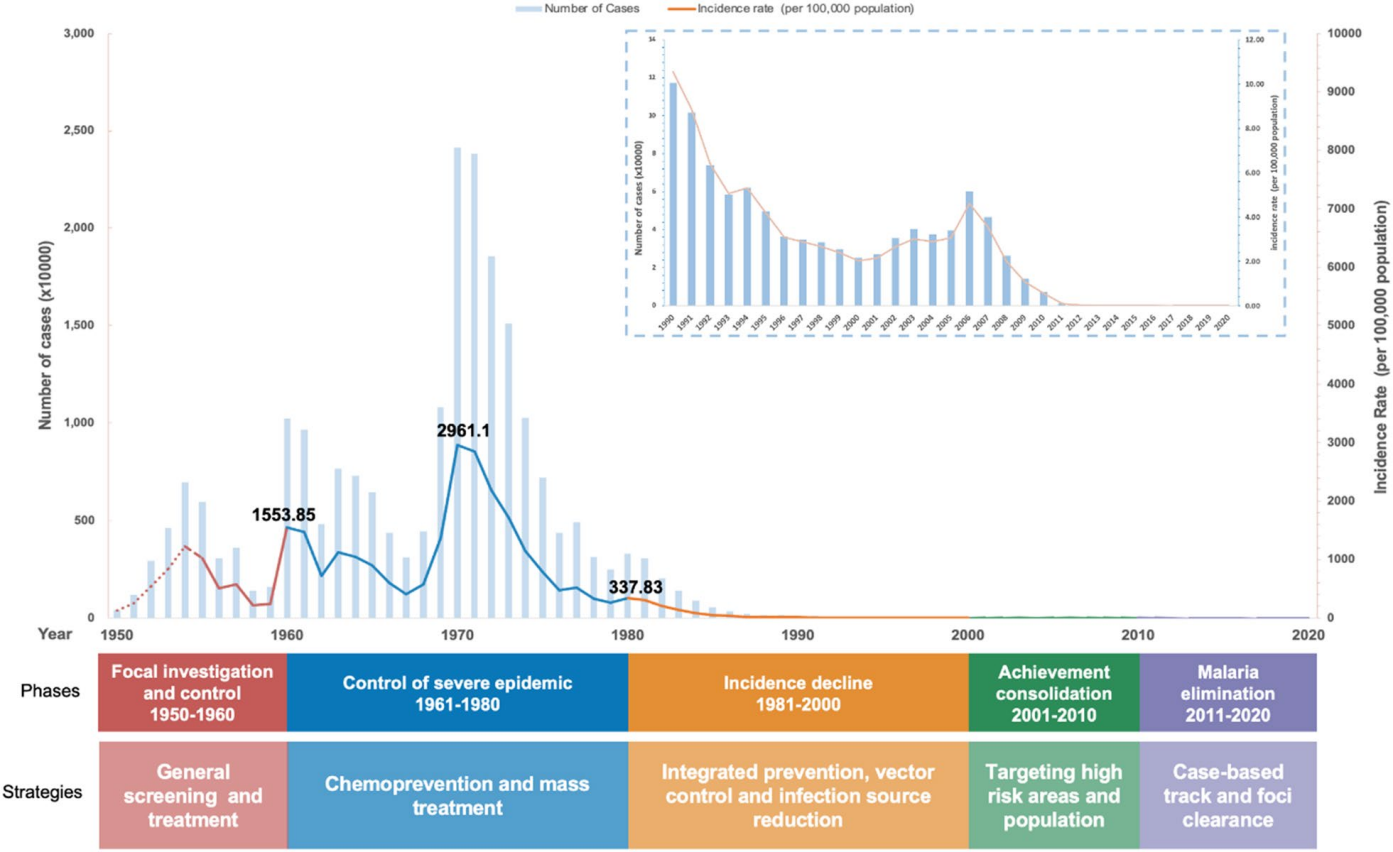
Five phases of China’s malaria control and elimination strategies, 1949–2020

Since the 1950s, China has transitioned from a malaria pandemic country with tens of millions of annual cases, through phases of local control and elimination, to sustained national elimination. This marks the first time a country in the World Health Organization (WHO) Western Pacific Region has been certified malaria-free in more than 3 decades [2]. Beneath this headline success story, however, China’s ‘long march’ to malaria elimination shows great variation and adaptiveness. The approaches that were appropriate in a low-income, predominantly rural and agricultural country in the 1950s and 1960s are not the same as those needed 50 years later, in a richer and increasingly urban country, with a different malaria profile (Fig. 1). Meanwhile, China’s size, and the variation in conditions across the country—in levels of economic and social development, resource availability, and technical capacity—militate against adopting one-size-fits-all solutions. So, what underlies China’s success in eliminating malaria in such a vast country? Is this simply a case of ‘Chinese exceptionalism’, or are there things other countries—and China’s domestic global health and development community—can take away from China’s story? Our argument is that we need to look beyond the specific technical approaches that were adopted at different times and in different places. There is a need to better understand the ways in which policies and approaches have been locally tailored—both over time and to varied local conditions—and the systems and capacities which have made that possible. Ongoing work by the authors is identifying a number of key factors: First is the pragmatic adaptiveness of policies and strategies—and implementation. Since China’s first national malaria control plan in 1956, national policy has been continually adjusted to take account of changing practices and priorities. This is institutionalized through a national expert committee that advises the Ministry of Health, and through ongoing evaluation of implementation and performance, and feedback to policy decision-makers. This has allowed the central government to adjust over time, for example changing the relative emphasis on environmental management vs. protective measures for exposed populations [3], or stratifying counties strategies based on local malaria incidence to allow more local-tailored implementation as China entered its elimination phase in 2010. However, just looking at national-level adaptation misses the flexibility local governments have had to adapt their implementation to local realities, leading to locally-tailored, pragmatic and adaptive approaches. Examples include the use of specific control measures to reduce malaria incidence along China’s porous border with Myanmar and to deal with imported cases [4], or the implementation of locally-tailored mass drug administration to contain malaria re-emergence in China’s Huang-Huai Plain in 2006 [5].

Second, China has invested in building systems and capacities to support the elimination of parasitic diseases, including malaria [6]. The clearest example is the National Institute of Parasitic Diseases (NIPD), Chinese Center for Disease Control and Prevention (China CDC), and the national malaria control and prevention network stretching down to the lowest levels of government was established during the 1960s. Malaria control was subsequently included in the work of the whole country CDCs system (including the National, Provincial, Prefecture, and County) down to very local levels. This has been supported by systematic training and capacity development and, latterly, reporting systems. These systems and capacities building have been crucial in providing support at multiple levels [7], including to local-level implementation, data collection and analysis, and by building the mechanism through which technical expertise and experience at the grassroots can feed into adaptive strategy and policy development.

Third, the country has both benefited from, and contributed to, global health cooperation on malaria elimination. As in other areas, China has leveraged collaborations with external agencies, such as the Global Fund to Fight AIDS, Tuberculosis and Malaria, to actively develop its domestic systems and capacities [8]. However, as China’s domestic malaria elimination efforts has progressed, so has global health collaboration, initially with neighboring countries in Southeast Asia and subsequently with some countries from Africa.

Since 2015, the NIPD has been collaborating on a malaria control project in Tanzania, the China–UK–Tanzania Pilot Project on Malaria Control, supported by the China–UK Global Health Support Programme (GHSP) funded by the UK Department for International Development. The pilot has adapted elements of China’s domestic experience, including 1,7‑malaria Reactive Community‑based Testing and Response (1,7‑mRCTR). This is a locally‑tailored approach, using existing health facility data and locally trained community health workers to conduct community level testing and treatment, and rapidly report malaria cases [9].

This is the first pilot project in which a Chinese agency has collaborated in on-the-ground implementation of a malaria control project overseas. It has shown encouraging results, reducing the malaria burden in the intervention areas by 81% [10]. Equally importantly, it has provided a platform for engagement between a Chinese technical agency and a counterpart in an African country and created space for mutual learning. Work underway by the authors is documenting how learning can help inform future cooperation.

As China’s engagement in global health increases—in malaria and more widely—there is a need to look closely at the country’s experience on multiple levels. There is clearly a ‘what?’ question—China’s experience shows that specific technical approaches are important—for example early use of bed nets, or setting demanding milestones for its case detection and reporting system. But as we have argued, there is also a ‘how?’ question—how malaria control and elimination journey have been managed and the approaches and tools that have underpinned adaptation, local tailoring of solutions, and provided expert input to implementation and to the policy process. As the global health community seeks to understand and learn from China’s progress in areas of public health, and as Chinese policy makers explore new options for China’s future global health cooperation, this ‘how?’ question will become increasingly relevant, including for the capacities and systems China needs as it contemplates a greater engagement in global health. Broadening debates and bringing in new analytical perspectives can help support mutual learning—and improved outcomes—between Chinese agencies and their overseas partners as China’s global health engagement increases.

## Data Availability

Not applicable.

## Abbreviations

WHO: World Health Organization
COVID-19: Coronavirus Disease 2019
NIPD: National Institute of Parasitic Diseases
China CDC: Chinese Center for Disease Control and Prevention
GHSP: Global Health Support Programme
1,7‑mRCTR: 1,7‑malaria Reactive Community‑based Testing and Response
